# Endometriosis and New-Onset Coronary Artery Disease in Taiwan: A Nationwide Population-Based Study

**DOI:** 10.3389/fmed.2021.619664

**Published:** 2021-06-25

**Authors:** Chun-Hui Wei, Renin Chang, Yu Hsun Wan, Yao-Min Hung, James Cheng-Chung Wei

**Affiliations:** ^1^Department of Obstetrics and Gynecology, Liouying Chi Mei Medical Center, Tainan, Taiwan; ^2^Department of Emergency Medicine, Kaohsiung Veterans General Hospital, Kaohsiung, Taiwan; ^3^Department of Medical Research, Chung Shan Medical University Hospital, Taichung, Taiwan; ^4^Department of Internal Medicine, Kaohsiung Municipal United Hospital, Kaohsiung, Taiwan; ^5^School of Medicine, National Yang Ming University, Taipei, Taiwan; ^6^College of Health and Nursing, Meiho University, Pingtung, Taiwan; ^7^Division of Allergy, Immunology and Rheumatology, Chung Shan Medical University Hospital, Taichung, Taiwan; ^8^Institute of Medicine, Chung Shan Medical University, Taichung, Taiwan; ^9^Graduate Institute of Integrated Medicine, China Medical University, Taichung, Taiwan

**Keywords:** coronary artery disease, endometriosis, cohort study, CAD, new-onset

## Abstract

Endometriosis (EM) with chronic inflammation may accelerate the progression of atherosclerosis. Currently, no large or randomized clinical studies have assessed the incidence of cardiovascular events in patients with endometriosis in Asia to investigate whether incident EM is associated with a higher risk of new-onset coronary artery disease (CAD). In this study of a nationwide cohort in Taiwan, we identified 13,988 patients with newly diagnosed EM from 1 January, 2000, through 31 December, 2012. EM and non-EM groups were matched by propensity score at a ratio of 1:1. Of a total 27,976 participants, 358 developed CAD. The incidence rate in the EM group was higher than that in the non-EM group (1.8 per 1,000 person-years vs. 1.3 per 1,000 person-years) during the follow-up period. The adjusted hazard ratio (aHR) of CAD for the EM group was 1.52 with a 95% confidence interval (1.23–1.87, *p* < 0.001) after adjusting for demographic characteristics, comorbidities, surgical procedures, frequency of outpatient visits, and medications. Stratified analysis revealed that, among four age groups (20–39, 40–49, 50–54, and above 55 years), the 20–39 years sub-group was associated with a higher risk of CAD (aHR, 1.73; 95% CI, 1.16–2.59, *p* = 0.008). Several sensitivity analyses were conducted for cross-validation, and it showed consistent positive findings. In conclusion, this cohort study revealed that patients with symptomatic EM in Taiwan were associated with increased risk of subsequent CAD than patients without medical records of EM. Further prospective studies are needed to confirm this causal relationship.

## Introduction

Endometriosis (EM) is a common estrogen-dependent gynecologic disorder that is prevalent in reproductive-aged women (1–3), and EM is defined as extrauterine endometrial glands and stromal growth primarily on ovaries and the pelvic peritoneum (2, 3). It occurs in about 8.9% of the general population in Taiwan (4). The prevalence of infertility in patients is as high as 70% and can reach 90% in women with chronic pelvic pain syndrome (5–7). Women with EM represent a high-risk population group for several co-morbidities of gynecological and non-gynecological diseases (8), including ovarian cancer and cardiovascular disorders with links to systemic chronic inflammation, elevated atherogenic lipid profile, and heightened oxidative stress (9). Previous studies have suggested that women with EM have elevated levels of interleukin-1, and interleukin-6. Tumor necrosis factor-α and markers of oxidants have been detected in the peritoneal fluid and peripheral blood of women in the EM group (10–13). Higher serum levels of low-density lipoprotein and lower high-density lipoprotein have also been found in women with EM (14–16). These are all risk factors that may accelerate the progression of atherosclerosis and contribute to the incidence of coronary artery disease (CAD), which can be fatal in advanced countries, including Taiwan. Although mortality rates decreased by as much as 50% in the 1990s and 2000s, over four million people have died annually from CAD in over 49 countries in Europe and North Asia (17).

However, epidemiologic evidence of the association between EM and CAD remains limited. A prospective cohort study focusing on a group of nurses in the USA was proposed (9). We conducted a large nationwide population-based cohort study to further explore the epidemiologic relationship between incident EM and the subsequent development of CAD in Taiwan's general female population.

## Methods

### Data Source

The data in this study were obtained from Taiwan's National Health Insurance Research Database (NHIRD), which contains healthcare data for more than 99% of residents in Taiwan since 1995. Within Taiwan's National Health Insurance (NHI) scheme, which is a universal health insurance program, all medical claims are mandatorily sent to the Bureau of National Health Insurance (BNHI) for validation and reimbursement. The NHIRD collects beneficiaries' registration files regarding demographics, all types of medical visits, laboratory test codes, procedure codes, prescription codes, and diagnostic codes based on the International Classification of Diseases, 9th Revision, Clinical Modification (ICD-9-CM) system. The Longitudinal Health Insurance Database 2000 (LHID 2000), a subset of NHIRD, was used in this study. There is no statistically significant difference in distributions in age, sex, or health care costs between the 1,000,000 people from the LHID 2000 and those in the original NHIRD. The original identification number of each patient in this data set was encrypted to protect privacy. The Institutional Review Board of Chung Shan Medical University in Taiwan approved this study (IRB permit number CS15134) and waived the need for informed consent, since the data were used anonymously and anonymized before analysis.

### Study Subjects and Study Design

**Figure 1:**
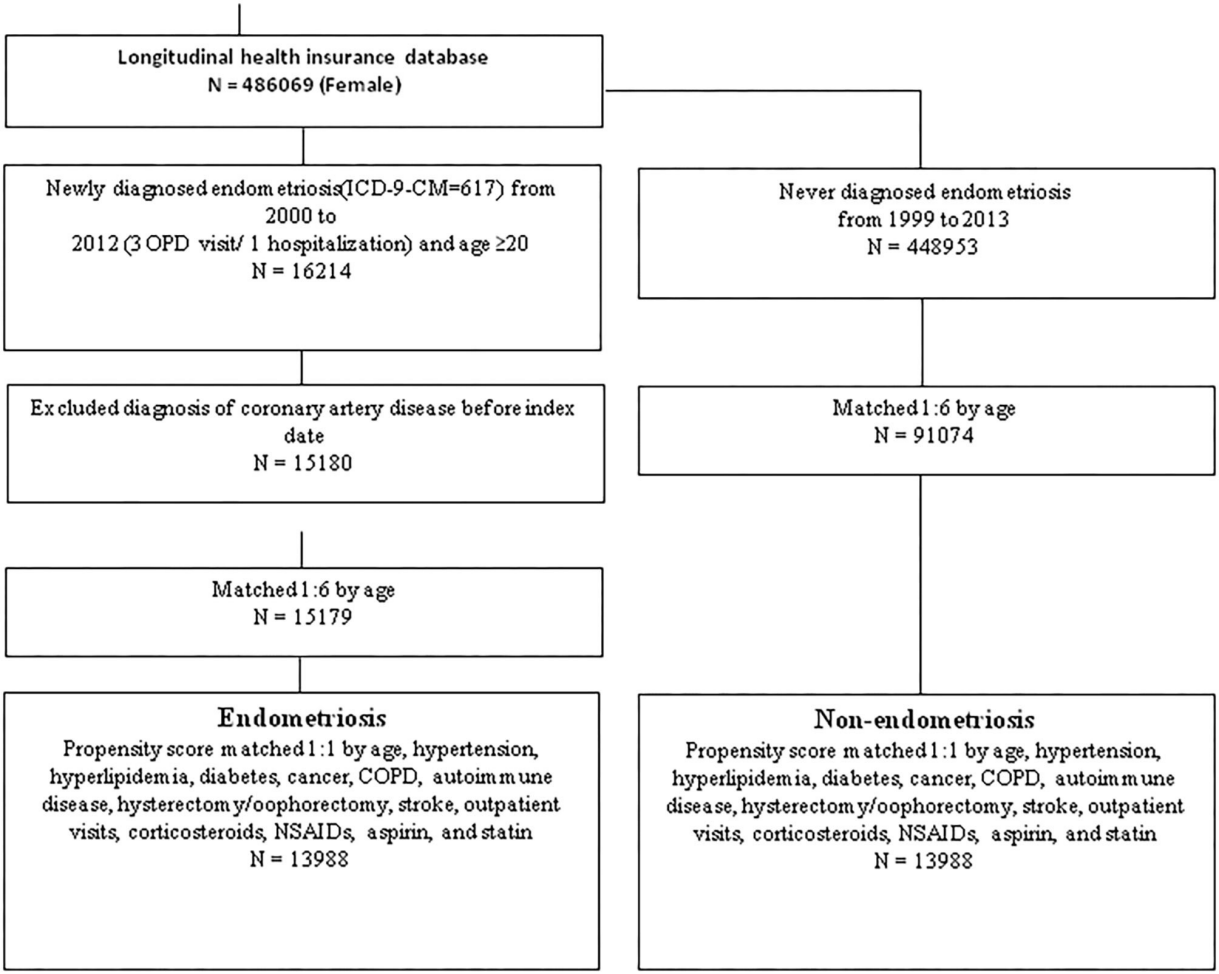
Flowchart of the retrospecive cohort study in Taiwan.

Study subjects were sampled from the LHID 2000 data. We identified patients newly diagnosed with endometriosis (ICD-9-CM codes 617) between January 2000 and December 2012 from both outpatient and inpatient visits (Figure 1). The index date was defined as the first date of EM diagnosis in either outpatient visit or inpatient admission. Only patients with at least 1 inpatient admission or 3 outpatient visits within 1 year before the index were enrolled. The exclusion criteria for the study subjects were: (1) patients diagnosed with EM before the index date, or during the follow-up period; (2) patients with a history of CAD (ICD-9-CM code 410–414) before the index date; (3) patients under 20 years old. As a result, a total of 27,976 eligible participants were identified from the LHID 2000 (13,988 in the EM group and 13,988 in the non-EM group). The control group was selected from the LHID 2000 using propensity scores matched at a ratio of 1:1 by age, sex, index date, comorbidities, surgical procedures, outpatient visits, and medications. The age of each study subject was determined using the difference between the index date and the date of birth. Subjects with a history of CAD who were diagnosed before the index date were excluded. Individuals in both the EM and non-EM groups were monitored until a CAD occurred or they were withdrawn from the national health insurance system, or until 31, December 2013.

### Outcome and Covariates

The outcome of this study was the incidence of newly diagnosed CAD in both the study and control groups. The definition of newly diagnosed CAD required (1) the presence of ICD-9-CM code 410–414; (2) emergency or inpatient diagnosis of CAD. The covariates compared in both the groups included the age at the index date, and baseline comorbidities such as hypertension (ICD-9-CM codes 401–405), hyperlipidemia (ICD-9-CM codes 272.0–272.4), diabetes (ICD-9-CM codes 250), cancer (ICD-9-CM codes 140–208), chronic obstructive pulmonary disease (ICD-9-CM codes 491, 492, 496; COPD), and autoimmune diseases (ICD-9-CM codes 710.0, 714.0, 720.0); surgical procedures such as hysterectomy and oophorectomy (ICD-OP-CODE 65.5, 65.6, 68.3–68.5, 68.9); stroke (ICD-9-CM codes 430–438); and co-medications including corticosteroids, non-steroidal anti-inflammatory drugs (NSAIDs), aspirin, and statins. The age of the subjects was categorized into four groups: 20–39, 40–49, 50–54, and over 55 years. The common comorbidities analyzed in this study were the medical disorders, namely, the risk factors associated with CAD. Information on comorbid medical disorders was obtained by tracing at least one inpatient admission or 3 outpatient visits in the NHI database within 1 year before the index date. Medication confounders in this study were corticosteroids, NSAIDs, aspirin, and statins.

### Propensity Score Matching

Because in age-matching (i.e., before propensity score matched) the EM group displayed a greater prevalence of comorbidities and therefore carried a greater risk of CAD than the non-EM group, propensity score matching was performed to balance the distribution of those potential risk factors (Table 1, right column). The propensity score was calculated using logistic regression to estimate the probability of EM, based on the baseline variables including age, hypertension, hyperlipidemia, diabetes, cancer, COPD, autoimmune disease, surgical procedures (hysterectomy/oophorectomy), stroke, frequency of outpatient visits, and co-medications including corticosteroids, non-steroidal anti-inflammatory drugs (NSAIDs), aspirin, and statins.

**Table 1:** Demographic characteristics of endometriosis and non-endometriosis.

	**Before propensity score matched**					**After propensity score matched**				
	**Endometriosis** **(*****N*** **=** **15,179)**		**Non-endometriosis** **(*****N*** **=** **91,074)**			**Endometriosis** **(*****N*** **=** **13,988)**		**Non-endometriosis** **(*****N*** **=** **13,988)**		
	***n***	**%**	***n***	**%**	***p-*value**	**n**	**%**	**n**	**%**	***p-*value**
Age			1					0.970		
20–39	8,170	53.8	49,020	53.8		7,970	57.0	7,967	57.0	
40–49	6,008	39.6	36,048	39.6		5,229	37.4	5,216	37.3	
50–54	779	5.1	4,674	5.1		640	4.6	657	4.7	
≥55	222	1.5	1,332	1.5		149	1.1	148	1.1	
Mean ± SD	38.4 ± 8.5		38.4 ± 8.5		1	37.8 ± 8.4		37.9 ± 8.5		0.104
Hypertension	600	4.0	2,584	2.8	<0.001	452	3.2	454	3.2	0.946
Hyperlipidemia	231	1.5	1,081	1.2	0.001	186	1.3	189	1.4	0.876
Diabetes	258	1.7	1,260	1.4	0.002	209	1.5	186	1.3	0.244
Cancer	325	2.1	742	0.8	<0.001	228	1.6	243	1.7	0.486
COPD	82	0.5	272	0.3	<0.001	71	0.5	66	0.5	0.668
Autoimmune disease	81	0.5	353	0.4	0.009	74	0.5	70	0.5	0.738
Hysterectomy/oophorectomy	2,654	17.5	1,481	1.6	<0.001	1,463	10.5	1,453	10.4	0.845
Stroke	483	3.2	2,210	2.4	<0.001	394	2.8	397	2.8	0.914
Outpatient visits	166.6 ± 140.8		115.4 ± 116		<0.001	164.3 ± 139		162.9 ± 133.2		0.407
Corticosteroids	9,169	60.4	48,396	53.1	<0.001	8,413	60.1	8,430	60.3	0.836
NSAIDs	14,928	98.3	82,953	91.1	<0.001	13,741	98.2	13,734	98.2	0.752
Aspirin	1,788	11.8	7,827	8.6	<0.001	1,550	11.1	1,557	11.1	0.894
Statin	1,651	10.9	7,702	8.5	<0.001	1,367	9.8	1,396	10.0	0.561

*COPD, Chronic obstructive pulmonary disease.*

*NSAIDs, Non-steroidal anti-inflammatory drugs*.

### Statistical Analysis

Some demographic characteristic data were analyzed using the Chi-square (χ2) test or Student's *t-*test, including age distributions, comorbidities, and medications between the EM group and the non-EM group. The incidence density of CAD per 1,000 person-years was calculated in both groups. To investigate the association between EM and CAD, a Cox proportional hazard regression analysis was conducted to measure the hazard ratios (HRs) and 95% confidence intervals (CIs) after adjusting for age, hypertension, hyperlipidemia, diabetes, cancer, COPD, autoimmune diseases, stroke, hysterectomy/oophorectomy, frequency of outpatient visits, corticosteroids, NSAIDs, aspirin, and statin. The Kaplan–Meier method was used to describe the cumulative incidence of CAD among the two groups. Differences were evaluated using the log rank test. Sub-group analyses were performed to identify the contribution of covariates.

### Inverse Probability of Treatment Weighting (IPTW)

We conducted a sensitivity analysis. Baseline differences were balanced by the inverse probability of treatment weighting (IPTW) based on the propensity score. The significance level was set as a 2-tailed *p-*value of 0.05. All data and statistics were processed and analyzed by SPSS version 18.0 (SPSS Inc., Chicago, IL, USA).

## Results

As shown in Figure 1, Taiwan's LHID enrolled a total of 486,069 female participants. After propensity score matching, this study included 13,988 EM patients and 13,988 non-EM patients registered between 1 January 2000, and 31 December 2012. A flowchart for participants' enrollment in the study is depicted in Figure 1. The baseline characteristics of the two groups are listed in Table 1, which shows that the distribution of covariates in matched pairs was similar and well-balanced after propensity score matched (right column). Both groups had similar frequencies of comorbidities, medication, outpatient clinic utility, and hysterectomy, and oophorectomy. The mean age of the EM group was 37.8 (±8.4) years.

Table 2 shows the results of Cox proportional hazard modeling for the evaluation of potential risk factors for developing CAD, including demographic characteristics, comorbidities, surgical procedures, and medications. The incidence rates of CAD in the EM and non-EM groups were 1.8 and 1.3 per 1,000 person-years, respectively. Compared to the non-EM group, the crude HR for CAD for the EM group was 1.46 (95% CI, 1.19–1.80, *p* < 0.001). The adjusted HR for CAD for the EM group was 1.52 (95% CI, 1.23–1.87, *p* < 0.001) in comparison with the non-EM group after adjusting for confounding factors.

**Table 2:** Cox proportional hazard model for estimation of adjusted HRs on coronary artery disease.

	**No. of CAD**	**Observed Person-Years**	**Incidence Density (Per 1,000 Person-Years)**	**Crude HR**	**95% C.I**.	***p-*value**	**Adjusted HR^**†**^**	**95% C.I**.	***p-*value**
Endometriosis									
No	159	122,678	1.3	1			1		
Yes	199	108,346	1.8	1.46	1.19–1.80	<0.001	1.52	1.23–1.87	<0.001
Age									
20–39	98	136,674	0.7	1			1		
40–49	209	83,225	2.5	3.60	2.83–4.58	<0.001	2.51	1.94–3.24	<0.001
50–54	36	9,095	4.0	5.97	4.07–8.76	<0.001	2.36	1.55–3.58	<0.001
≥55	15	2,031	7.4	11.00	6.39–18.95	<0.001	3.14	1.74–5.66	<0.001
Hypertension	53	6,210	8.5	6.66	4.97–8.92	<0.001	1.90	1.37–2.65	<0.001
Hyperlipidemia	14	2,467	5.7	4.05	2.37–6.92	<0.001	1.12	0.63–2.00	0.695
Diabetes	29	2,740	10.6	7.76	5.3–11.34	<0.001	2.58	1.68–3.97	<0.001
Cancer	6	3,194	1.9	1.28	0.57–2.87	0.548	0.96	0.42–2.17	0.915
COPD	3	1,204	2.5	1.59	0.51–4.95	0.425	1.52	0.48–4.77	0.476
Autoimmune disease	4	1,039	3.8	2.60	0.97–6.97	0.057	2.04	0.76–5.49	0.160
Hysterectomy/oophorectomy	89	22,713	3.9	3.09	2.43–3.92	<0.001	1.42	1.10–1.84	0.007
Stroke	58	7,344	7.9	5.70	4.30–7.55	<0.001	2.11	1.56–2.86	<0.001
Outpatient visits	–	–	–	0.999	0.998–0.9998	0.014	0.996	0.995–0.997	<0.001
Corticosteroids	209	154,386	1.4	0.64	0.52–0.79	<0.001	0.67	0.54–0.84	<0.001
NSAIDs	354	228,782	1.5	0.74	0.28–2.00	0.557	1.01	0.37–2.75	0.983
Aspirin	215	30,318	7.1	9.55	7.73–11.81	<0.001	9.44	7.45–11.96	<0.001
Statin	112	26,296	4.3	3.40	2.72–4.26	<0.001	1.35	1.04–1.75	0.023

† *Adjusted for age, hypertension, hyperlipidemia, diabetes, cancer, COPD, autoimmune disease, hysterectomy/oophorectomy, stroke, outpatient visits, corticosteroids, NSAIDs, aspirin, and statin.*

*COPD, Chronic obstructive pulmonary disease.*

*NSAIDs, Non-Steroidal Anti-Inflammatory Drugs*.

**Figure 2:**
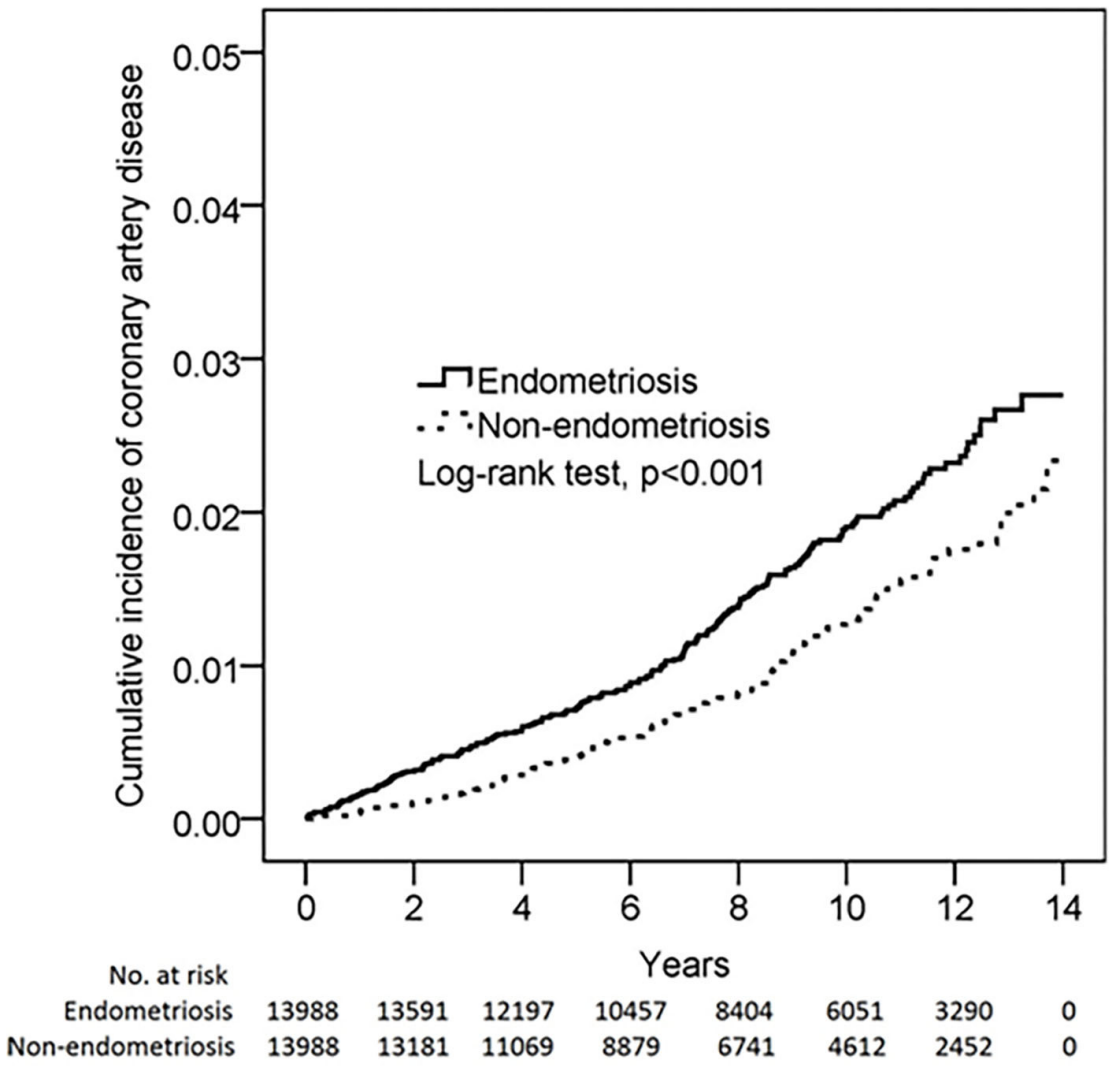
The Kaplan-Meier curves of incidence of CAD in subjects with and without EM.

Figure 2 shows the Kaplan-Meier curves of incidence of CAD in subjects with and without EM.

**Figure 3:**
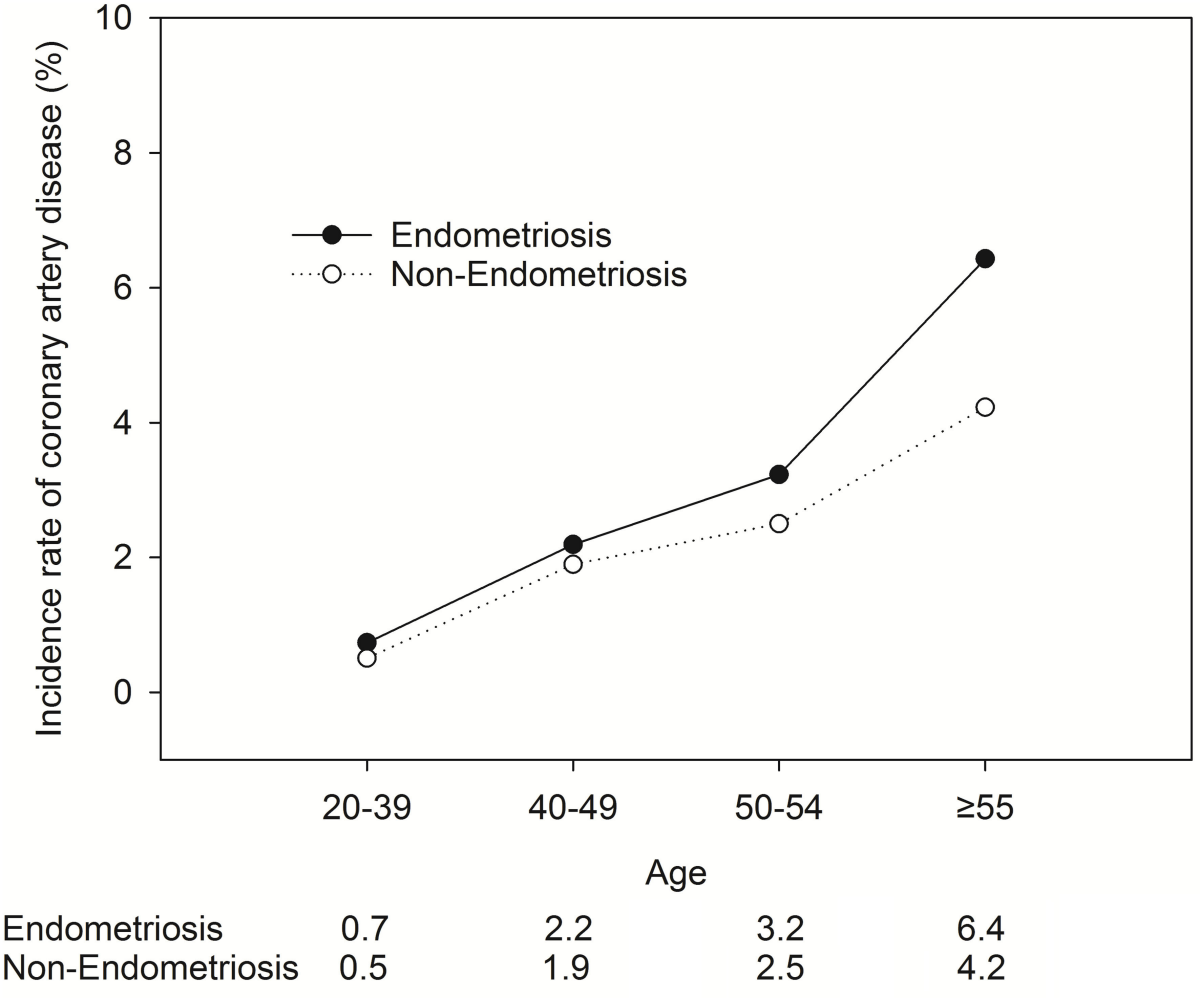
The relationship between the CAD incidence rate (Y-axis) and age (X-axis) among individuals with or without EM.

Figure 3 shows the relationship between the CAD incidence rate (Y-axis) and age (X-axis) among individuals with or without EM for additional insights.

Table 3 displays the hazard ratios and the 95% confidence intervals of CAD risk using stratified Cox proportional hazard regression among the matched groups. Stratified analysis revealed that the adjusted HR was 1.73 (95% CI = 1.16–2.59, *p* = 0.008) in the subgroup of patients aged 20–39 years. The adjusted HR was 1.33 (95% CI = 1.02–1.75, *p* = 0.039) in a subgroup of patients from 40 to 49 years old. The *P-*value for interaction was 0.785. There seemed to be no interaction effects between age and the exposure of EM on the incidence of subsequent CAD.

**Table 3:** Subgroup analysis of Cox proportional hazard model.

	**Endometriosis**		**Non-endometriosis**				
	***N***	**No. of CAD**	***N***	**No. of CAD**	**HR**	**95% CI**	***p-*value**
Age							
20–39	7,970	58	7,967	40	1.73	1.16–2.59	0.008
40–49	5,229	112	5,216	97	1.33	1.02–1.75	0.039
50–54	640	20	657	16	1.50	0.78–2.90	0.228
≥55	149	9	148	6	1.67	0.59–4.68	0.333
						p for interaction = 0.785	
Hypertension							
No	13,536	171	13,534	134	1.50	1.20–1.88	<0.001
Yes	452	28	454	25	1.27	0.74–2.17	0.392
						p for interaction = 0.592	
Hyperlipidemia							
No	13,802	191	13,799	153	1.46	1.18–1.81	<0.001
Yes	186	8	189	6	1.55	0.54–4.47	0.418
						p for interaction = 0.859	
Diabetes							
No	13,779	184	13,802	145	1.49	1.20–1.85	<0.001
Yes	209	15	186	14	1.06	0.51–2.20	0.875
						p for interaction = 0.376	
Cancer							
No	13,760	196	13,745	156	1.47	1.19–1.82	<0.001
Yes	228	3	243	3	1.01	0.20–5.02	0.987
						p for interaction = 0.672	
COPD							
No	13,917	197	13,922	158	1.46	1.19–1.80	<0.001
Yes	71	2	66	1	1.90	0.17–20.94	0.601
						p for interaction = 0.785	
Autoimmune disease							
No	13,914	196	13,918	158	1.45	1.18–1.79	0.001
Yes	74	3	70	1	3.88	0.40–37.77	0.243
						p for interaction = 0.442	
Hysterectomy/oophorectomy							
No	12,525	152	12,535	117	1.56	1.23–1.99	<0.001
Yes	1,463	47	1,453	42	1.10	0.73–1.67	0.644
						p for interaction = 0.161	
Stroke							
No	13,594	170	13,591	130	1.53	1.22–1.92	<0.001
Yes	394	29	397	29	1.16	0.69–1.94	0.574
						p for interaction = 0.330	
Corticosteroids							
No	5,575	87	5,558	62	1.70	1.22–2.35	0.001
Yes	8,413	112	8,430	97	1.34	1.02–1.76	0.036
						p for interaction = 0.199	
NSAIDs							
No	247	2	254	2	1.04	0.15–7.41	0.965
Yes	13,741	197	13,734	157	1.47	1.19–1.81	<0.001
						p for interaction = 0.819	
Aspirin							
No	12,438	87	12,431	56	1.79	1.28–2.50	0.001
Yes	1,550	112	1,557	103	1.23	0.94–1.60	0.137
						p for interaction = 0.068	
Statin							
No	12,621	138	12,592	108	1.48	1.15–1.90	0.002
Yes	1,367	61	1,396	51	1.37	0.94–1.98	0.100
						p for interaction = 0.634	

*Hazard ration (HR) was estimated by univariate Cox proportional hazard model.*

*COPD, Chronic obstructive pulmonary disease.*

*NSAIDs, Non-Steroidal Anti-Inflammatory Drugs*.

Tables 4, 5 provide two sensitivity analyses examining the reliability of the HR of CAD in different matching methods and covariate adjustment. Table 4 shows that for the sensitivity analysis using IPTW, the adjusted HR was 1.55 (95% CI = 1.44–1.67, *p* < 0.001). Table 5 shows that for another sensitivity analysis using IPTW with different scenarios (adjusted age, comorbidities, surgical procedures, corticosteroids, and NSAIDs (not including the protective medications aspirin and statins), the adjusted HR was 1.51 (95% CI = 1.39–1.62, *p* < 0.001).

**Table 4:** Sensitivity analysis by using inverse probability of treatment weighting.

	***N***	**No. of CAD**	**Crude HR**	**95% C.I**.	***p-*value**	**Adjusted HR^**†**^**	**95% C.I**.	***p-*value**
Endometriosis								
No	91,074	1,013	1			1		
Yes	15,179	234	1.40	1.30–1.51	<0.001	1.55	1.44–1.67	<0.001

† *Adjusted for age, hypertension, hyperlipidemia, diabetes, cancer, COPD, autoimmune disease, hysterectomy/oophorectomy, stroke, outpatient visits, corticosteroids, NSAIDs, aspirin, and statin*.

**Table 5:** Sensitivity analysis by using inverse probability of treatment weighting (not included aspirin and statin).

	***N***	**No. of CAD**	**Crude HR**	**95% C.I**.	***p-*value**	**Adjusted HR^**†**^**	**95% C.I**.	***p-*value**
Endometriosis								
No	91,074	1,013	1			1		
Yes	15,179	234	1.40	1.30–1.51	<0.001	1.51	1.39–1.62	<0.001

† *Adjusted for age, hypertension, hyperlipidemia, diabetes, cancer, COPD, autoimmune disease, hysterectomy/oophorectomy, stroke, outpatient visits, corticosteroids, and NSAIDs*.

In Supplement Table 1, we have added a subgroup analysis. We applied hormone therapy as a proxy for severer EM. Detail information about hormone therapy is listed in Supplement Table 2. Our findings show that among EM patients, the risk of subsequent CAD was higher in the hormone therapy subgroup than in non-EM patients (aHR, 2.01; 95% CI, 1.59–2.54, *P* < 0.001). We hypothesize that EM patients treated with hormones indicate a more severe EM status.

In Supplement Table 3, we have added a sensitivity analysis for a different scenario to define EM cases. Since the gold standard diagnosis of EM is based on the laparoscopy, and may be validated by more advanced image system, such as magnetic resonance image, therefore, eligible EM patients were defined as ICD coding with associated examinations (such as laparoscopy, or magnetic resonance image) which was performed within 180 days before or after the index date. The result shows consistent finding that EM is positively associated with subsequent CAD (aHR, 1.60; 95% CI, 1.29–1.98, *P* < 0.001).

## Discussion

The results of this nationwide population-based cohort study showed that patients with a history of EM were associated with a 1.52-fold risk of new-onset CAD compared with the general population without a medical diagnosis of EM. Furthermore, associations of EM with CAD were found to be most significant in younger female patients in the 20–39 years age group. Atherosclerosis is a progressive disease that may take years to advance. Therefore, the separations of two KM curves in the study just after the diagnosis of EM may be not fully explained by chronic inflammation. Notably, indolent EM has an average period of 8–10 years before clinical diagnosis in Asia and Europe (18).

The study results suggest that EM is a potential risk factor for CAD. Gynecologists tend to ignore the possible high risk of CAD among EM patients. The comprehensive treatment plan provided to people with traditional CAD risk factors should also be provided to patients with EM, especially young women. Policy makers are encouraged to enforce screening for CADs in endometriosis patients aged 20–39 years and to provide more integrated care between gynecology, cardiology, and healthy lifestyle promotion.

This study had several strengths, including longitudinal study design, large sample size, 13-year follow-up, good sampling method, and data obtained from a nationwide population-based dataset involving a whole country with one single ethnic population, rather than purposive sampling (only registered female nurses or specific age group). The propensity score matching also balanced the distribution of potential risk factors between the two groups, enabling comparison.

The advantages of using the NHIRD in epidemiological research have previously been described in detail (19). The large sample size allowed us to perform subgroup analysis and illustrate the interactions of different age groups and comorbidities. A previous large cohort study in the USA Nurses' Health Study II with 116,430 women reported that laparoscopic-confirmed endometriosis was associated with a higher risk of CAD. The strength of the NHS II study is the prospective design, laparoscopy and angiography confirmation of disease status, and inclusion of additional CAD risk factors such as lifestyle, family history, and anthropometric measurements. The association was stronger among young women (under the age of 54). In addition, it has been mentioned that the treatments for endometriosis, such as hysterectomy or oophorectomy, revealed an increased risk of CAD in women with EM (9). However, in our subgroup analysis, patients with EM and a related surgical procedure (hysterectomy or oophorectomy) did not have a higher risk of CAD (aHR 1.10, 95% CI = 0.73–1.67, *p-*value for interaction = 0.161). We speculate that surgically treated EM patients may be accompanied by a regression of ED in association with chronic inflammation and a reduced risk of subsequent development of CAD. Furthermore, based on our findings, although surgical treatment of EM may affect normal ovarian function, in general it should not be considered to lead to vulnerability to CAD. Further studies are required to examine the effect of surgical treatments on EM and subsequent development of CAD. The participants in (9) included different ethnic backgrounds and the diagnosis of non-fatal myocardial infarction events was from self-reported questionnaire feedback. Therefore, the real incidence of CAD events in the patients with EM could not be assessed.

Nevertheless, this study proposed that the increased risk of CAD in the women with EM was significant especially in women of the 20–39 years age group. This study also demonstrated that EM was an independent risk factor for developing CAD in Asian women aged 20–39, which was different from the study in USA. As no similar study had previously been conducted on the Asian population, possible causes might include racial, and life-style factors or other unmeasured confounders that were not available in the dataset of this study. A prominent risk of developing CAD was only observed in the first 3 years after the EM diagnosis, which might be the result of diminished inflammation associated with atherosclerosis. The patients' inflammatory status was relieved because of the medications and treatment they had undergone since the diagnosis. The underlying mechanism through which EM increases the risk of developing CAD may be the synergistic effects of systemic chronic inflammation, heightened oxidative stress, and atherogenic lipid profile associated with EM. In addition, it has been mentioned that EM, and CAD may share common genetic susceptibilities (9).

Several limitations regarding the analysis of the results need to be stated. First, the ICD-9-CM codes for the diagnosis of EM, CAD, and medical comorbidities were based on administrative claim data recorded by physicians and hospitals instead of a chart review. Consequently, there might be some inaccuracy that could have resulted in misclassification, although the Bureau of NHI uses an auditing mechanism to minimize diagnostic uncertainty and misclassification (20). EM may not be detected in most patients, and there could be under-reporting of cases. However, such non-differential misclassification always swayed the results toward the null (21). Second, some potential confounding factors of CAD like obesity, smoking, physical activity, and degree of systemic inflammation (like C-reactive protein) were not covered in this study, although COPD had been investigated in several previous studies (22, 23) as a proxy variable for cigarette smoking. Third, it remains uncertain whether the findings in this study could be generalized to other ethnic groups. The findings, therefore, should be interpreted with caution given some methodological flaws, such as the absence of data on important CAD risk factors and EM disease severity measurement.

## Conclusions

This 13-year national population-based cohort study showed that patients with EM were associated with a higher incidence of CAD after adjusting for demographic characteristics, relevant confounding factors. Further studies are encouraged to focus on EM treatment, including anti-estrogen effect, or androgen like drug treatment or transient decline of estrogen after definite surgery, and subsequent risk of CAD.

## Data Availability Statement

The original contributions presented in the study are included in the article/Supplementary Material, further inquiries can be directed to the corresponding author/s.

## Ethics Statement

The studies involving human participants were reviewed and approved by The Institutional Review Board of Chung Shan Medical University in Taiwan approved this study (IRB permit number CS15134) and waived the need for informed consent, since the data were used anonymously and anonymized before analysis. Written informed consent for participation was not required for this study in accordance with the national legislation and the institutional requirements.

## Author Contributions

C-HW, RC, and Y-MH: conceptualization. YHW: data curation, formal analysis, funding acquisition, and software. C-HW: investigation. C-HW, RC, YHW, Y-MH, and JW: methodology. JW: resources. Y-MH and JW: supervision. C-HW and RC: writing–original draft preparation. RC and Y-MH: writing–review and editing. All authors were involved in drafting the article or revising it and all authors approved the final version to be published.

## Conflict of Interest

The authors declare that the research was conducted in the absence of any commercial or financial relationships that could be construed as a potential conflict of interest.

## References

[1] Choi EJ Cho SB Lee SR Lim YM Jeong K Moon HS. Comorbidity of gynecological and non-gynecological diseases with adenomyosis and endometriosis. Obstet Gynecol Sci. (2017) 60:579–86. 10.5468/ogs.2017.60.6.579 29184867 PMC5694733

[2] Giudice LC Kao LC. Endometriosis. Lancet. (2004) 364:1789–99. 10.1016/S0140-6736(04)17403-5 15541453

[3] Bulun SE. Endometriosis. N Engl J Med. (2009) 360:268–79. 10.1056/NEJMra0804690 19144942

[4] Teng SW Horng HC Ho CH Yen MS Chao HT Wang PH. Women with endometriosis have higher comorbidities: analysis of domestic data in Taiwan. J Chin Med Assoc. (2016) 79:577–82. 10.1016/j.jcma.2016.04.006 27553580

[5] Practice Committee of the American Society for Reproductive Medicine. Treatment of pelvic pain associated with endometriosis: a committee opinion. Fertil. Steril. (2014) 101:927–35. 10.1016/j.fertnstert.2014.02.012 24630080

[6] Hemmings R Rivard M Olive DL Poliquin-Fleury J Gagné D Hugo P. Evaluation of risk factors associated with endometriosis. Fertil Steril. (2004) 81:1513–21. 10.1016/j.fertnstert.2003.10.038 15193470

[7] Renner SP Strick R Fasching PA Oeser S Oppelt P Mueller A. Single nucleotide polymorphisms in the progesterone receptor gene and association with uterine leiomyoma tumor characteristics and disease risk. Am J Obstet Gynecol. (2008) 199:648.e1–9. 10.1016/j.ajog.2008.06.015 18691687

[8] Matalliotaki C Matalliotakis M Zervou MI Trivli A Matalliotakis I Mavromatidis G. Co-existence of endometriosis with 13 non-gynecological co-morbidities: Mutation analysis by whole exome sequencing. Mol Med Rep. (2018) 18:5053–7. 10.3892/mmr.2018.9521 30272298 PMC6236265

[9] Mu F Rich-Edwards J Rimm EB Spiegelman D Missmer SA. Endometriosis and risk of coronary heart disease. Circ Cardiovasc Qual Outcomes. (2016) 9:257–64. 10.1161/CIRCOUTCOMES.115.002224 27025928 PMC4940126

[10] Agic A Xu H Finas D Banz C Diedrich K Hornung D. Is endometriosis associated with systemic subclinical inflammation? Gynecol Obstet Invest. (2006) 62:139–47. 10.1159/000093121 16679772

[11] Bedaiwy MA Falcone T Sharma RK Goldberg JM Attaran M Nelson DR. Prediction of endometriosis with serum and peritoneal fluid markers: a prospective controlled trial. Hum Reprod. (2002) 17:426–31. 10.1093/humrep/17.2.426 11821289

[12] Akoum A Al-Akoum M Lemay A Maheux R Leboeuf M. Imbalance in the peritoneal levels of interleukin 1 and its decoy inhibitory receptor type II in endometriosis women with infertility and pelvic pain. Fertil Steril. (2008) 89:1618–24. 10.1016/j.fertnstert.2007.06.019 17919610

[13] Koumantakis E Matalliotakis I Neonaki M Froudarakis G Georgoulias V. Soluble serum interleukin-2 receptor, interleukin-6 and interleukin-1a in patients with endometriosis and in controls. Arch Gynecol Obstet. (1994) 255:107–12. 10.1007/BF02390936 7979562

[14] Verit FF Erel O Celik N. Serum paraoxonase-1 activity in women with endometriosis and its relationship with the stage of the disease. Hum Reprod. (2008) 23:100–4. 10.1093/humrep/dem340 18000171

[15] Melo AS Rosa-e-Silva JC Rosa-e-Silva AC Poli-Neto OB Ferriani RA Vieira CS. Unfavorable lipid profile in women with endometriosis. Fertil Steril. (2010) 93:2433–6. 10.1016/j.fertnstert.2009.08.043 19969295

[16] Turgut A Özler A Görük NY Tunc SY Evliyaoglu O Gül T. Copper, ceruloplasmin and oxidative stress in patients with advanced-stage endometriosis. Eur Rev Med Pharmacol Sci. (2013) 17:1472–8. 23771536

[17] Benjamin EJ Virani SS Callaway CW Chamberlain AM Chang AR Cheng S. Heart disease and stroke statistics-2018 update: a report from the American heart association. Circulation. (2018) 137:e67–492. 10.1161/CIR.0000000000000558 29386200

[18] Parasar P Ozcan P Terry KL. Endometriosis: epidemiology, diagnosis and clinical management. Curr Obstet Gynecol Rep. (2017) 6:34–41. 10.1007/s13669-017-0187-1 29276652 PMC5737931

[19] Hsing AW Ioannidis JP. Nationwide population science: lessons from the Taiwan national health insurance research database. JAMA Intern Med. (2015) 175:1527–9. 10.1001/jamainternmed.2015.3540 26192815

[20] Cheng TM. Taiwan's new national health insurance program: genesis and experience so far. Health Aff . (2003) 22:61–76. 10.1377/hlthaff.22.3.61 12757273

[21] Copeland KT Checkoway H McMichael AJ Holbrook RH. Bias due to misclassification in the estimation of relative risk. Am. J. Epidemiol. (1977) 105:488–95. 10.1093/oxfordjournals.aje.a112408 871121

[22] Chang KH Chang MY Muo CH Wu TN Chen CY Kao CH. Increased risk of dementia in patients exposed to nitrogen dioxide and carbon monoxide: a population-based retrospective cohort study. PLoS ONE. (2014) 9:e103078. 10.1371/journal.pone.0103078 25115939 PMC4130523

[23] Raaschou P Simard JF Asker Hagelberg C Askling J. Rheumatoid arthritis, anti-tumour necrosis factor treatment, and risk of squamous cell and basal cell skin cancer: cohort study based on nationwide prospectively recorded data from Sweden. BMJ. (2016) 352:i262. 10.1136/bmj.i262 26823527 PMC4730989

