# Supplementary material for: Endometriosis and New-Onset Coronary Artery Disease in Taiwan: A Nationwide Population-Based Study
Source: Front Med (Lausanne). 2021 Jun 25;8:619664. doi: 10.3389/fmed.2021.619664 (PMC8290915; doi:10.3389/fmed.2021.619664)
Supplement: Supplementary file 1 [file Data_Sheet_1.docx]

**Supplement table 1**

|  | N | No. of CAD | Crude HR | 95% C.I. | p-value |  | Adjusted HR^†^ | 95% C.I. | p-value |
| --- | --- | --- | --- | --- | --- | --- | --- | --- | --- |
| Endometriosis |  |  |  |  |  |  |  |  |  |
| Non-EM | 13988 | 159 | 1 |  |  |  | 1 |  |  |
| EM without hormonal treatment | 7138 | 68 | 1.02 | 0.77-1.35 | 0.894 |  | 1.03 | 0.77-1.37 | 0.843 |
| EM with hormonal treatment | 6850 | 131 | 1.89 | 1.5-2.39 | <0.001 |  | 2.01 | 1.59-2.54 | <0.001 |

†Adjusted for age, hypertension, hyperlipidemia, diabetes, cancer, COPD, autoimmune disease, hysterectomy/oophorectomy, stroke, outpatient visits, corticosteroids, NSAIDs, aspirin, and statin.

Use of medication was defined as the prescription for at least 30 days of drug use within 180 days before or after the index date.

**Supplement table 2**

**Hormonal medications Anatomical Therapeutic Chemical Classification System codes:**

G02BA Intrauterine Contraceptives

G03A Hormonal Contraceptives For Systemic Use

G03CA Natural And Semisynthetic Estrogens, Plain

G03CB Synthetic Estrogens, Plain

G03CX Other Estrogens

G03DA02 Medroxyprogesterone

G03DA03 Hydroxyprogesterone

G03DA04 Progesterone

G03DB08 Dienogest

G03DC02 Norethisterone

G03FA01 Norethisterone And Estrogen

G03FA02 Hydroxyprogesterone And Estrogen

G03FA04 Progesterone And Estrogen

G03FA11 Levonorgestrel And Estrogen

G03FA12 Medroxyprogesterone And Estrogen

G03FA15 Dienogest And Estrogen

G03FB05 Norethisterone And Estrogen

G03FB06 Medroxyprogesterone And Estrogen

G03FB09 Levonorgestrel And Estrogen

G03GA Gonadotropins

G03HB01 Cyproterone And Estrogen

G03XA Antigonadotropins And Similar Agents

G03XB Progesterone Receptor Modulators

G03XC Selective Estrogen Receptor Modulators

H01CA Gonadotropin-Releasing Hormones

H01CC Anti-Gonadotropin-Releasing Hormones

**Supplement table 3** (Stricter definition for endometriosis)

|  | N | No. of CAD | Crude HR | 95% C.I. | p-value |  | Adjusted HR^†^ | 95% C.I. | p-value |
| --- | --- | --- | --- | --- | --- | --- | --- | --- | --- |
| Endometriosis |  |  |  |  |  |  |  |  |  |
| Non-EM | 13988 | 159 | 1 |  |  |  | 1 |  |  |
| EM with associated examination | 12266 | 186 | 1.56 | 1.26-1.93 | <0.001 |  | 1.60 | 1.29-1.98 | <0.001 |

†Adjusted for age, hypertension, hyperlipidemia, diabetes, cancer, COPD, autoimmune disease, hysterectomy/oophorectomy, stroke, Outpatient visits, corticosteroids, NSAIDs, aspirin, and statin.

EM associated examinations are defined performed within 180 days before or after the index date.

EM associated examinations included any of the following:

28014C Laparoscopy

19003C Gynecological ultrasonography

33084/33085 Magnetic resononace image
